# Assessing Gender Differences in Technical Skills and Confidence in Orthopaedic Surgery Residency Applicants

**DOI:** 10.5435/JAAOSGlobal-D-22-00265

**Published:** 2023-07-06

**Authors:** Jona Kerluku, Lauren Wessel, Daphne Ling, Joseph T. Nguyen, Karla J. Felix, Karen M. Sutton, Duretti T. Fufa

**Affiliations:** From the Department of Hand and Upper Extremity Surgery, Hospital for Special Surgery, New York, NY (Ms. Kerluku and Dr. Fufa); the Department of Orthopaedic Surgery, University of California Los Angeles, Los Angeles, CA (Dr. Wessel); the Department of Orthopedics, Chang Gung Memorial Hospital, Taoyuan, Taiwan (Dr. Ling); the Department of Population Health Sciences, Weill Cornell Medical College, New York, NY (Dr. Ling); the HSS Research Institute, Hospital for Special Surgery, New York, NY (Dr. Ling); the HSS Research Institute, Biostatistics Core, Hospital for Special Surgery, New York, NY (Mr. Nguyen); the Department of Academic Training, Hospital for Special Surgery, New York, NY (Dr. Felix); and the Department of Orthopaedic Surgery, Sports Medicine, Hospital for Special Surgery, New York, NY (Dr. Sutton).

## Abstract

Variations in confidence for procedural skills have been demonstrated when comparing male and female medical students in surgical training. This study investigates whether differences in technical skill and self-reported confidence exist between male and female medical students applying to orthopaedic residency.

**Methods:**

All medical students (2017 to 2020) invited to interview at a single orthopaedic residency program were prospectively evaluated on their technical skills and self-reported confidence. Objective evaluation of technical skill included scores for a suturing task as evaluated by faculty graders. Self-reported confidence in technical skills was assessed before and after completing the assigned task. Scores for male and female students were compared by age, self-identified race/ethnicity, number of publications at the time of application, athletic background, and US Medical Licensing Examination Step 1 score.

**Results:**

Two hundred sixteen medical students were interviewed, of which 73% were male (n = 158). No gender differences were observed in suture task technical skill scores or mean difference in simultaneous visual task scores. The mean change from pre-task and post-task self-reported confidence scores was similar between sexes. Although female students trended toward lower post-task self-reported confidence scores compared with male students, this did not achieve statistical significance. Lower self-reported confidence was associated with a higher US Medical Licensing Examination score and with attending a private medical school.

**Discussion:**

No difference in technical skill or confidence was found between male and female applicants to a single orthopaedic surgery residency program. Female applicants trended toward self-reporting lower confidence than male applicants in post-task evaluations. Differences in confidence have been shown previously in surgical trainees, which may suggest that differences in skill and confidence may develop during residency training.

Confidence is crucial for the development of decision-making capability in surgery. General surgery residents and attendings rated competence and confidence as essential traits during critical situations in the operating room.^1^ Differences between male and female confidence in performing procedural skills are reported as early as medical school.^5,6^ Overall, medical schools offer highly variable experiences in exposure to basic procedural skills. In efforts to standardize student competence regarding basic procedural skills required for graduation, medical schools facilitate student exposure in low-risk simulated environments.^5-10^ One study demonstrated that male medical students had, on average, markedly more experience at each level of learning with essential bedside procedures and were markedly more confident than female students.^5^ In surgical boot camps designed for fourth-year medical students, male and female students earned similar objective scores, yet female medical students reported lower baseline self-efficacy scores.^6^

Developing trainee abilities to practice independence remains a priority, even in learning environments where medical professionals face increased stress, often because of medicolegal concerns and documentation requirements, all while managing responsibilities to optimize patient outcomes.^3,4,22^ Surgeon educators monitor medical knowledge, technical performance, and individual resident characteristics to determine the appropriate level of autonomy trainees are granted during a procedure.^23^ Lack of confidence in technical skills was identified as a “red flag,” often serving as a cue for attending surgeons to take over a case.^21-23^

Although these associations are difficult to study, with few objective measurements of technical skill, it is possible that perceptions of a resident's skills, preparedness, and confidence can be influenced by implicit bias. Previous studies of general and thoracic surgery training programs have demonstrated that female trainees are less likely to be left alone in the operating room and less likely to be given the lead during cases.^13,20^ Even after controlling for difficulty of cases, faculty sex, resident training level, and case volume, results from seven academic medical centers found sex to be an independent predictor for surgical room autonomy.^13^ Differences in technical skill were not assessed in these studies, and the reasons for these differences remain poorly understood; however, the fact that female students are given less independence may play a role.

In this study, objective measures of technical skill and confidence in technical skill were assessed to investigate whether there are differences between male and female students interviewed by a single orthopaedic residency program. We sought to test the null hypothesis that no differences in objective or perceived skill would exist between male and female students.

## Methods

All medical students from 2017 to 2020 who were invited to interview at a single accredited orthopaedic residency training program were evaluated after institutional review board approval. Interviewee age, race/ethnicity, sex, number of peer-reviewed publications at the time of application, athletic background (yes/no), and US Medical Licensing Examination (USMLE) Step 1 score were recorded. Sex and race/ethnicity were self-reported by interviewees and designated according to their Association for American Medical Colleges application. Athletic background was accounted for in applicants reporting a collegiate or professional athletic history in their activities section or personal statement. Study procedures followed Association for American Medical College data policy guidelines for applicant confidentiality.

### Objective Evaluation of Technical Skill

**Figure 1:**
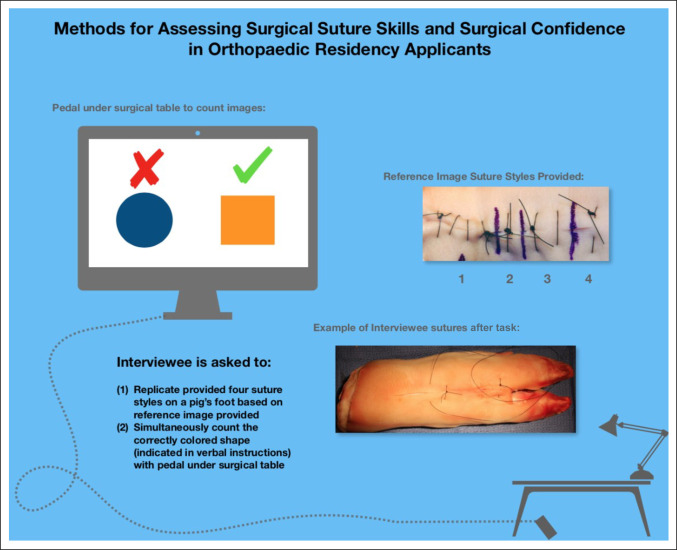
Illustration showing methods assessing surgical suture skills and surgical confidence during interviews of orthopaedic surgery residency applicants.

All interviewees were evaluated for technical skill in two ways: (1) direct observation by board-certified orthopaedic surgeons achieving a single consensus score on performance of a timed, simulated suturing task (scale 1-3) and (2) accuracy of performance of a simultaneous visual field task (Figure 1). There was no instruction given before the interview day. The visual task involved preassigned on-screen images of colored shapes for the applicant to manually count using a foot pedal under the surgical table. The correct pedal count (preassigned based on-screen images) and total applicant pedal count were recorded to measure the ability to multitask. Standardized instructions were provided to each interviewee immediately before beginning the 8-minute task.

### Self-Reported Confidence in Technical Skill

Before and after completing the assigned task, interviewees were asked to self-evaluate their confidence in technical skills (scale 1 to 10, with 10 representing the most confident). Individual scores for technical skill and self-reported confidence in technical skills were compared by self-reported sex of the interviewee. Pre-test and post-test scores were evaluated using multivariable regression to determine demographic factors influencing self-reported confidence. The analysis of covariance model was used to see how scores differed between male and female interviewees after adjusting for the other variables used in the previous regression model (including pre-task and post-task evaluations). Statistical significance was defined as *P*-values of 0.05 or less. All data were analyzed using SPSS version 23.0 (IBM).

## Results

Data for 216 orthopaedic surgery interviewees were reviewed, including 158 male students (73%). The average ages of female and male students were 26.7 (range, 24 to 33) and 26.9 (range, 23 to 32) years, respectively. Eighty-one percent of female students (n = 47) and 74% of male students (n = 117) who were invited to interview attended a private medical school. Sixty-two percent of female students (n = 36) and 58% of male students (n = 91) reported a previous collegiate or professional athletic history in their orthopaedic residency application. None of these differences reached statistical significance (Table 1). Male students invited to interview had a significantly greater number of average peer-reviewed journal publications at the time of application (10.7 vs. 6.8, *P* = 0.01). Male students also had a higher average USMLE Step 1 score (254 vs. 251, *P* = 0.04).

**Table 1:** Student and Application Demographics

	Male Mean or % (SD or N)	Female Mean or % (SD or N)	*P* Value
	73(158)	27(58)	—
Race/ethnicity			0.83
White	64(101)	55(32)	—
Asian	19(30)	22(13)	—
Hispanic or Latino	6(10)	9(5)	—
Black or African American	6(9)	7(4)	—
Other	5(8)	7(4)	—
Age	26.9(1.9)	26.7(1.5)	0.77
Completed clinical rotation at the interviewing Institution	59(94)	64(37)	0.57
Collegiate athlete history	58(91)	62(36)	0.55
Private medical school	74(117)	81(47)	0.29
Peer-reviewed journal publications	10.7(10.9)	6.8(7.2)	0.01
USMLE Step 1 score	254.1(9.4)	251.1(9.6)	0.04
USMLE Step 2 score	260.1(10.3)	262.5(8.4)	0.28

USMLE = US Medical Licensing Examination.

**Figure 2:**
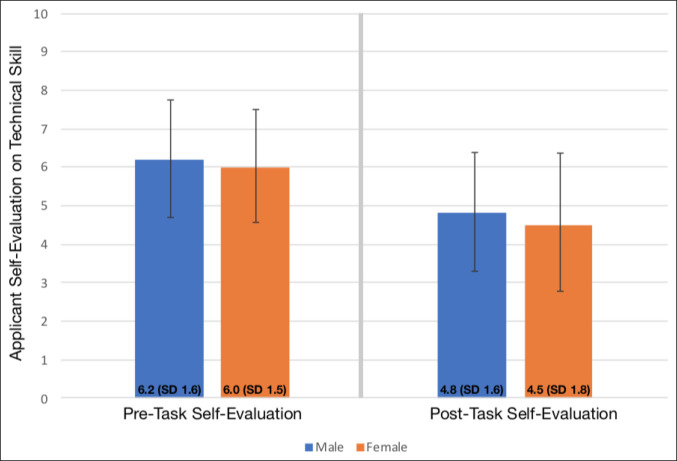
Graph showing applicant self-evaluation scores on technical performance before and after the task based on sex.

No significant differences by sex were found in objective evaluation of technical skill for the simulated suturing task (female 1.7 versus male 1.7, *P* = 0.77) or for the simultaneous visual field task represented by the difference between correct pedal count and total interviewee pedal count (female 2.7 versus male 3.2, *P* = 0.41) (Table 2). Both female and male students significantly changed to lower confidence self-evaluation scores on post-task performance compared with pre-task performance (*P* < 0.001 for both groups; Figure 2). No statistically significant mean change between female and male students from before the task to after it was determined for self-evaluation scores (*P* = 0.91). Although female students scored themselves lower than male students on both the pre-task and post-task evaluation scores, in the multivariable regression model, these differences were not statistically significant (*P* = 0.24 and *P* = 0.08, respectively). Private medical school education and higher Step 1 scores (*P* = 0.03) were associated with lower pre-task and post-task self-reported confidence scores (Tables 3 and 4).

**Table 2:** Univariable Regression Model Assessing Simulated Suture Task and Simultaneous Visual Field Task Differences Between Female and Male Students

	Student Cohort	Male Mean (SD)		Female Mean (SD)		*P* Value
Simulated suture task	1.7 (0.6)	1.7 (0.6)		1.7 (0.6)		0.77
		**Interviewee** **Pedal count**	**Correct** **Pedal count**	**Interviewee** **Pedal count**	**Correct** **Pedal count**	
Simultaneous visual field task	3.0 (4.2)^a^	9.0 (4.5)	12.2 (1.4)	9.5 (3.9)	12.2 (1.5)	0.41^a^

a Refers to difference between correct pedal count and applicant pedal count.

**Table 3:** Multivariable Regression Model Assessing Student Pre-task Self-Reported Confidence During Orthopaedic Residency Interviews

Outcome	Variable	Beta	Standard Error	95% Confidence Interval		*P* Value
				Lower	Upper	
Pre-task self-rated confidence scores	Intercept	4.36	5.04	—	—	—
	Female (versus male)	−0.42	0.36	−1.13	0.29	0.24
	Age	0.17	0.09	−0.01	0.35	0.06
	Previous HSS rotation	0.21	0.32	−0.43	0.84	0.52
	Private medical school (versus public)	−0.69	0.33	−1.35	−0.03	**0.04**
	Previous collegiate athlete	−0.49	0.34	−1.17	0.19	0.15
	Peer-reviewed journal publication	0.00	0.02	−0.03	0.04	0.85
	USMLE Step 1 score	−0.05	0.02	−0.09	0.00	**0.03**
	USMLE Step 2 score	0.04	0.02	−0.01	0.08	0.11

HSS = Hospital for Special Surgery, USMLE = US Medical Licensing Examination. Bolded values indicate statistical significance at the p<0.05 level.

**Table 4:** Multivariable Regression Model Assessing Student Post-task Self-Reported Confidence During Orthopaedic Residency Interviews

Outcome	Variable	Beta	Standard Error	95% Confidence Interval		*P* Value
				Lower	Upper	
Post-task self-rated confidence scores	Intercept	6.32	6.22	—	—	—
	Female (versus male)	−0.79	0.45	−1.68	0.10	0.08
	Age	0.09	0.11	−0.13	0.32	0.42
	Previous HSS rotation	0.18	0.40	−0.60	0.97	0.65
	Private medical school (versus public)	0.05	0.42	−0.78	0.88	0.90
	Previous collegiate athlete	−0.02	0.42	−0.86	0.82	0.96
	Peer-reviewed journal publication	0.02	0.02	−0.02	0.06	0.27
	USMLE Step 1 score	−0.06	0.03	−0.11	−0.01	**0.03**
	USMLE Step 2 score	0.04	0.03	−0.02	0.10	0.16

HSS = Hospital for Special Surgery, USMLE = US Medical Licensing Examination. Bolded values indicate statistical significance at the p<0.05 level.

## Discussion

No difference in objective evaluation of technical skill was found between male and female medical students applying to a single orthopaedic surgery residency program. Student self-reported confidence in technical skill markedly decreased between pre-task and post-task self-reporting. Although female students self-reported lower confidence in technical skill, this did not reach statistical significance. Similarly, a 2021 study of surgical trainees demonstrated that there was no difference in attending ratings of surgical performance for female trainees compared with male trainees.^20^ However, they found that female trainees rated themselves as having less autonomy and worse performance than male trainees when controlling for training level, attending, procedure, case complexity, and attending ratings.^20^ Lower levels of confidence among female surgical trainees have been previously reported in the field of general surgery. In a survey of 4136 surgical trainees, Bucholz et al^17^ identified lower levels of surgical confidence among female trainees. These confidence levels also persist past the training period. In this study, female trainees were twice as likely to worry about competence after training. Although the methodology to assess confidence between the present study and other discussed studies is variable, given that we did not reveal confidence differences in medical students, these findings may suggest that differences may be compounded during residency training.

Lees et al identified surgical experience as a critical factor in the development of confidence.^11^ Other studies reviewed resident self-evaluations of laparoscopy simulation and showed that women underestimate their performance while men overestimate their performance.^15^ Female residents also exhibit greater cautiousness in avoiding errors.^11,15,16^ In a study that investigated the barriers and facilitators of resident autonomy, vascular surgery program directors, compared with trainees, were markedly more likely to think that an attending should take over the case if trainees were unsure of themselves or if surgical room efficiency was compromised.^12,14^ Taken together, these studies suggest how differences can potentially develop in a training setting in which a subset of trainees have less autonomy. While the longer term effect of these perhaps subtle differences is not well-understood, lower confidence and diminished self-efficacy has been suggested to lead to greater levels of stress and subsequent burnout, which have also been noted at greater levels within the female surgeon population.^2,12^ As such, it is important to carefully consider how surgical educators can improve the consistency of surgical opportunity and objective feedback to improve trainee experiences in procedural training.^18^

We did find an association between USMLE Step 1 score and private medical school training and lower self-reported confidence scores. These findings are difficult to explain, especially with USMLE Step 1 transitioning to a pass/fail reporting.^24^ We can hypothesize that these differences may be secondary to differential procedural exposure of applicants during medical school training, which may be associated with institutional practices. Several studies have compared medical student procedural exposure during clinical rotations to self-reported confidence levels while performing technical skills.^5,10,19^ Experience with procedural skills during medical school is important toward achieving competency before graduation; however, studies show great variability in medical student experiences, although applicants to surgical residencies were not found to have markedly more procedural exposure.^5,10,19^ Therefore, our findings of lower self-reported confidence with students coming from private medical schools and achieving higher USMLE Step 1 scores may be an outcome of changes in clinical medical curriculum or changes in medical student integration with clinical teams during rotations and may be a subject for future investigation.

Our study has limitations. This study reflects a specific group of medical students who earned an interview at a highly academic, urban orthopaedic residency program, so results may not be representative of all medical students applying to orthopaedic surgery. While the surgical skill task used was objective and applied consistently, it is not a validated task and our scoring system scale of 1 to 3 may not have been sensitive enough to determine differences between the groups. In addition, given that measurement of skill was done at a single time point, we cannot account for a student's prior experience with the task or its stability over time. Owing to the demographics of medical students who apply to orthopaedic surgery residencies, our study population was skewed toward male students and, therefore, could have been underpowered to detect a gender difference, particularly given that the post-task confidence scores trended toward being lower in female students. This is a common limitation of single-institutional investigations, and future research can improve on this work through multi-institutional assessments. Furthermore, applicant interpretation of self-rated confidence questions could have influenced responses given the several tasks occurring in an interview setting. Incorporating existing validated short self-efficacy surveys in future investigations would improve this response bias, in addition to potentially creating new validated tools that could be used during the orthopaedic residency interview process. Finally, while the purpose of this study was to determine whether differences in technical skill and confidence exist, our ability to discuss the future implications of these differences relies solely on other literature because this study population was not studied in an ongoing fashion.

In conclusion, this study found no notable differences in technical skill or confidence between male and female orthopaedic surgery applicants. Given that previous studies have demonstrated sex-based differences in surgical trainees may suggest that differences develop throughout residency training which may be linked to perceived or actual differences in the learning environment experienced by male and female trainees. Additional research is required to better understand how technical skill and confidence may differ between male and female students and develop throughout orthopaedic surgery residency training.
